# Reduced morphine consumption and pain intensity with local infiltration analgesia (LIA) following total knee arthroplasty: A randomized double-blind study involving 48 patients

**DOI:** 10.3109/17453674.2010.487241

**Published:** 2010-05-21

**Authors:** Per Essving, Kjell Axelsson, Jill Kjellberg, Örjan Wallgren, Anil Gupta, Anders Lundin

**Affiliations:** ^1^Department of Orthopedic Surgery; ^2^Department of Anesthesiology and Intensive Care; ^3^Department of Physiotherapy, Division of Clinical Medicine, University HospitalÖrebro; ^4^University of Linköping, LinköpingSweden

## Abstract

**Background and purpose:**

Postoperative pain is often severe after total knee arthroplasty (TKA). We investigated the efficacy of the local infiltration analgesia (LIA) technique, both intraoperatively and postoperatively.

**Methods:**

48 patients undergoing TKA were randomized into 2 groups in a double-blind study. In group A, 400 mg ropivacaine, 30 mg ketorolac, and 0.5 mg epinephrine were infiltrated periarticularly during operation. In group P, no injections were given. 21 h postoperatively, 200 mg ropivacaine, 30 mg ketorolac, and 0.1 mg epinephrine were injected intraarticularly in group A, and the same volume of saline was injected in group P. All patients were followed up for 3 months.

**Results:**

Median morphine consumption was lower in group A during the first 48 h: 18 (1–74) mg vs. 87 (36–160) mg in group P. Postoperative pain was lower at rest in group A during the first 27 h, and on movement during the first 48 h, except at 21 h. Time to fulfillment of discharge criteria was shorter in group A than in group P: 3 (1–7) vs. 5 (2–8) days. Patient satisfaction was higher in group A than in group P on days 1 and 7. The unbound venous blood concentration of ropivacaine was below systemic toxic blood concentrations.

**Interpretation:**

The local infiltration analgesia (LIA) technique provides excellent pain relief and lower morphine consumption following TKA, resulting in shorter time to home readiness and higher patient satisfaction. There were few side effects and systemic LA concentrations were low.

## Introduction

Postoperative pain is usually severe after knee arthroplasty (Wang et al. 2002). In an attempt to improve pain relief, a local infiltration analgesia (LIA) technique was recently developed by Drs Kerr and Kohan in Sydney, Australia (Kerr and Kohan 2008). A long-acting local anesthetic (LA; ropivacaine), a nonsteroidal anti-inflammatory drug (ketorolac), and epinephrine are infiltrated periarticularly during operation and via an intraarticular catheter postoperatively.

In the last few years, several studies supporting the benefits of the LIA technique or the modified LIA technique in knee and hip arthroplasties have been published (Lombardi et al. 2004, Reilly et al. 2005, Andersen et al. 2007a, b, Parvataneni et al. 2007, Essving et al. 2009). However, there have only been 4 randomized controlled trials involving total knee arthroplasty (Busch et al. 2006, Vendittoli et al. 2006, Toftdahl et al. 2007, Andersen et al. 2008). Despite the great variability in reported performance of the LIA technique, all studies found that there was effective postoperative analgesia comparable with the standard for pain relief in TKA, the peripheral nerve block (Toftdahl et al. 2007). In contrast to the results of Kerr and Kohan, however, there was no evidence of any shortening of the hospital stay.

We have previously reported on a double-blind RCT on unicompartmental knee arthroplasty (UKA) performed with minimally invasive technique, using the LIA technique by Kerr and Kohan peri- and postoperatively (Essving et al. 2009). We found that there was a shorter hospital stay, and lower morphine consumption and pain intensity compared to placebo. Total knee arthroplasty (TKA) involves a greater degree of surgical trauma than UKA performed using the minimally invasive technique, resulting in more severe pain intensity. We were therefore interested in assessing the efficacy of the LIA technique during TKA.

The main purpose of the present study was therefore to evaluate whether the LIA technique would reduce morphine consumption during the first 48 postoperative hours. Secondary endpoints were pain intensity, time to home readiness using well-defined discharge criteria, side effects, plasma concentrations of local anesthetics, knee function, and patient satisfaction.

## Patients and methods

The study protocol was approved by the regional ethics committee (September 6, 2006; Dnr 2006/211) and the Swedish Medical Products Agency, and was conducted in accordance with the Declaration of Helsinki. It was also registered with ClinicalTrials.gov (identifier: NCT00799175).

78 consecutive patients scheduled for total knee arthroplasty (TKA) because of osteoarthritis were screened for eligibility. The inclusion criteria were: age 20–85 years, ASA I–III, and normal preoperative mobility. Exclusion criteria included known allergy or intolerance to one of the study drugs, serious liver-, heart- or renal disease, inflammatory joint disease, chronic pain, or any bleeding disorder.

### Randomization and blinding

**Figure 1.**
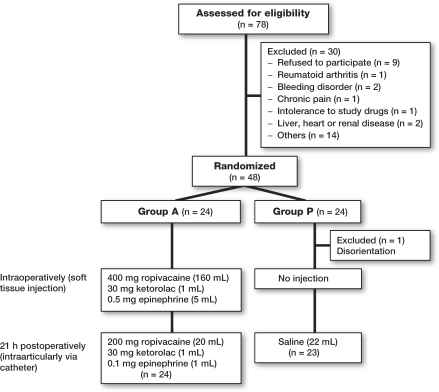
Flow chart for the study.

Of the 78 patients assessed for eligibility, 30 were excluded prior to randomization; see flow chart for details (Figure 1). Written informed consent was obtained from each patient before the start of the study. Surgery was performed at the Department of Orthopedics, Örebro University Hospital during April 2007 through September 2008 and patients were followed up for 3 months after surgery.

The hospital pharmacy randomized the patients into 2 groups with 24 patients in each, using computer-generated randomized numbers. Group A (Active) received a multimodal injection intra- and postoperatively while group P (Placebo) did not receive any injection intraoperatively and received a saline injection postoperatively as detailed below. On the day before or on the morning of surgery, the surgeon called the hospital pharmacy to receive the group randomization. The patients, the 2 study investigators, the study physiotherapist, and all the staff concerned with the postoperative care of the patients were blinded to the group randomization. Since the operating surgeons were not blinded, they did not take any part in the patient care after completion of the operation.

### Anesthesia

All patients received diazepam (10 mg) orally 1 h before planned surgery and all operations were performed under general anesthesia. Cloxacillin (1 g) was given intravenously before surgery and at 8, 16, and 24 h postoperatively. Dalteparin (5,000 IU) was administered subcutaneously once each evening for 10 days, starting on the evening before surgery.

### Surgery

All operations were performed using a standard medial parapatellar approach. All patients received an AGC prosthesis (Biomet, Warsaw, IN). A tourniquet was used in all patients and no drains were left in the knee joint after the operation. A compression bandage and ice packs were applied around the knee joint during the first 6 h.

### Pain management

In group A, 400 mg ropivacaine, 30 mg ketorolac, and 0.5 mg epinephrine (total volume 166 mL) were infiltrated by the surgeon into the soft tissues periarticularly during the operation in the following way: 300 mg ropivacaine, 30 mg ketorolac, and 0.5 mg epinephrine were mixed together to a total volume of 116 mL. Before inserting the prosthesis, 40–50 mL of the solution was injected into the posterior capsule. After the prosthesis was cemented in place, the rest of the solution was injected into the deep tissues around the ligaments, the capsule incision, and the synovium. In this way, all tissues that were traumatized received the analgesic solution. Before closing the skin, 50 mL ropivacaine (100 mg) without epinephrine or ketorolac was injected into the subcutaneous tissue. In group P, no injections were given. All patients had a tunnelled intraarticular multihole 20-G catheter placed at the end of the operation by the surgeon. A patient-controlled analgesia (PCA) pump with morphine (1-mg bolus and 6-min lockout time) was connected intravenously, which was used as rescue medication by all patients. All patients received 1 g paracetamol orally 4 times a day, starting on the morning of the operation. After 21 h, 200 mg ropivacaine, 30 mg ketorolac, and 0.1 mg epinephrine in total volume of 22 mL were injected intraarticularly via the catheter in group A and a similar volume of saline was injected in group P. Pain assessments were made by using a 100-mm VAS. At 24 h, if pain at rest was VAS < 40 mm over a 2-h period, the PCA pump was discontinued and paracetamol (1 g) and tramadol (100) mg orally were given up to 4 times daily as required. The intraarticular catheter was removed after 24 h and the tip of the catheter was sent for culture.

### Mobilization and home discharge

The first attempt at mobilization was made on the first postoperative morning, 1 h after the intraarticular injection. The patients were encouraged to stand up and to walk 2–3 steps, and they were discharged when they fulfilled the discharge criteria (see below). Following discharge home, all patients were asked to complete a questionnaire regarding postoperative pain on days 1, 3, and 14.

### Outcome measures: primary endpoint

#### Analgesic consumption

PCA-morphine consumption was recorded during 0–24, 24–48, and 0–48 h postoperatively. Oral analgesic consumption (tramadol) was recorded during 0–24, 24–48, and 0–48 h. Total analgesic consumption 0–48 h postoperatively was calculated by converting oral tramadol to the equivalent dose of intravenous morphine, i.e. 100 mg tramadol orally was equivalent to 10 mg morphine intravenously (Silvasti et al. 2000).

### Outcome measures: secondary endpoints

#### Pain

Pain assessment (VAS) was made preoperatively and at 3, 6, 12, 21, 22 (i.e. 1 h after test drug injection in the knee catheter), 27, and 48 h, and also on days 3 and 14, and at 3 months postoperatively. Pain was assessed both at rest and on flexion of the knee by 60 degrees.

#### Hospital stay

The time to fulfillment of discharge criteria (home readiness) was recorded by a physician and the study physiotherapist who were unaware of the group randomization. The discharge criteria were: mild pain (VAS < 30 at rest) sufficiently controlled by oral analgesics, ability to walk with elbow crutches, ability to climb 8 stairs, ability to eat and drink, and no evidence of any surgical complications. Length of hospital stay (LOS) was recorded as actual time to home discharge once the home discharge criteria were fulfilled (day 0 = the day of operation). Differences between the two could result from delayed discharge for administrative or social reasons.

#### Surgical outcome

Maximum knee extension and flexion were assessed preoperatively, at 24 h, 48 h, on day 3, at discharge, and after 3 months postoperatively. Timed up and go (TUG) test (Podsiadlo and Richardson 1991) was assessed preoperatively and postoperatively after 48 h, on days 3, 7 and 14, and after 3 months. Values of < 20 seconds indicate that the patient is indepentently mobile. An evaluation of patient satisfaction was done using a 4-grade verbal rating scale (excellent = 4, good = 3, inadequate = 2, poor = 1) during the first 24 postoperative hours and after 7 days. Oxford knee score was determined preoperatively, and at 2 weeks and 3 months postoperatively. Oxford knee score is a validated 12-item knee questionnaire that scores patients from 12 (the best possible) to 60 (the worst possible) (Jahromi et al. 2004). EuroCol (EQ-5D) questionnaire was collected preoperatively and postoperatively at 3 months. EuroCol (EQ-5D) is a standardized instrument for use as a measure of health outcome (Fransen and Edmonds 1999). It provides a single index value from 0 to 1 where 0 represents poor health and 1 represents perfect health.

#### Adverse effects

All complications and adverse events were registered intraoperatively and postoperatively, and also after discharge. Any hospital re-admissions during the 3-month follow-up period postoperatively were also recorded.

### Plasma concentrations of ropivacaine

In a sub-study in 8 patients, done prior to the main study, the LIA technique was performed in a similar way to that described earlier. Venous blood (7 mL) was taken in heparinized tubes postoperatively after 30, 60, 90, 120, and 180 min and just before and after the catheter injection at 30, 60, 90, 120, and 180 min. Total and free concentrations of ropivacaine were analyzed using chromatography with a Zorbax SB-C18 column.

### Statistics

A power analysis was done before the start of the study using morphine consumption over 48 hours postoperatively as the primary endpoint. In an earlier study on total knee arthroplasties (Axelsson et al. 2005), the mean morphine consumption over 48 hours postoperatively was 64.6 (SD 36.3) mg in the placebo group. The aim of this study was to investigate whether the LIA technique could reduce the morphine consumption by 50% to 32 mg. With an a of 0.05 and b of 0.2, we calculated that 24 patients would be required in each group if a non-parametric method was used. The number of patients for the sub-study was calculated to be 8.

The Mann-Whitney U test was used for the analysis of the primary endpoint (morphine consumption) since we found that the data were not normally distributed. Mann-Whitney U test was used to assess pain scores and the Bonferroni-Holm method was used to correct for multiple measures. Hospital stay, time to fulfill discharge criteria, knee function scores, and patient satisfaction scores were also analyzed using the Mann-Whitney U test. Dichotomous data were analyzed using the chi-squared test or Fisher's exact test, as appropriate. Values of p < 0.05 were considered to be statistically significant.

## Results

### Patients

One of the 48 randomized patients in group P became disoriented postoperatively and could not continue the study. Thus, 47 of the 48 randomized patients completed the study. Patient characteristics were similar in both groups (Table 1).

**Table 1.** Demografic data and duration of surgery. Values are mean (SD)

	Group A	Group P
No. of females/males	13/11	13/11
Age, years	72 (9)	70 (9)
Weight, kg	82 (14)	81 (10)
Height, cm	168 (9)	169 (8)
Body mass index	29 (5)	28 (3)
ASA, I / II / III	7 / 17 / 0	5 / 18 / 1
Operation time, min	93 (20)	87 (19)

Group A (active): intraoperative and postoperative injections. Group P (placebo): no intraoperative or postoperative injections. ASA physical status I: normal health; II: systemic disease with no limited activity; III: systemic disease with limited activity.

### Primary endpoint

Analgesic consumption. Median morphine consumption during the first 48 h postoperatively was lower in group A than in group P: 18 (1–74) mg vs. 87 (36–160) mg (p < 0.001), i.e. there was a median difference of 69 (95% CI: 47–86) mg (Table 2). The proportion of patients who requested ≥ 5 mg morphine during the first 24 h was significantly less in group A than in group P (0/23 vs. 10/24) (p < 0.01). Median total analgesic consumption (tramadol + morphine) during the first 48 postoperative hours was 54 (4–114) mg and 109 (37–221) mg, respectively (p < 0.001).

**Table 2.** Consumption of analgesics

	Group A median (range)	Group P median (range)	p-value
Morphine i.v. (mg)			
0–24 h	17 (1–74)	65 (36–131)	< 0.001
24–48 h	0.5 (0–17)	22 (0–52)	< 0.001
0–48 h	18 (1–74)	87 (36–160)	< 0.001
Tramadol orally (mg)			
0–24 h	0 (0–200)	0 (0–100)	0.01
24–48 h	375 (0–400)	200 (0–400)	0.04
0–48 h	400 (0–500)	200 (0–500)	0.008
Total analgesics ^**a**^ (mg)			
0–48 h	54 (4–114)	109 (37–211)	< 0.001

^**a**^ Total analgesic consumption was calculated by converting oral tramadol to the equivalent dose of intravenous morphine (100 mg tramadol orally = 10 mg morphine intravenously).

### Secondary endpoints

#### Pain relief

**Figure 2.**
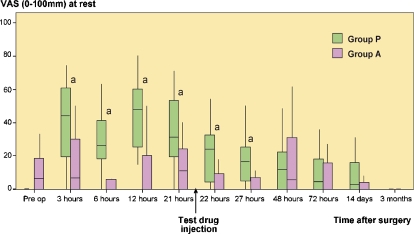
Postoperative pain at rest. VAS scores are presented as median and interquartile range (IQR). ^a^ p < 0.001 (3, 6, and 12 h); p = 0.005 (21 h); p = 0.003 (22 h); p = 0.002 (27 h).

At rest, median VAS pain score was statistically significantly lower in group A than in group P at 3, 6, 12, 21, 22, and 27 h (Figure 2).

**Figure 3.**
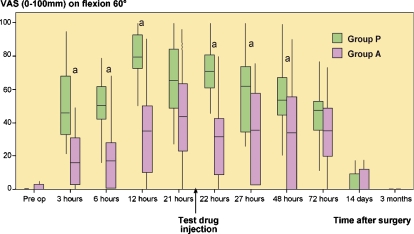
Postoperative pain on movement. VAS scores are presented as median and interquartile range (IQR). ^a^ p < 0.001 (3, 6, 12, and 22 h); p = 0.005 (27 h); p = 0.01 (48 h).

On movement, median VAS pain score was statistically significantly lower in group A than in group P at 3, 6, 12, 22, 27, and 48 h (Figure 3).

#### Hospital stay

Median time to home readiness was shorter in group A than in group P, 3 (1–7) vs. 5 (2–8) days (p = 0.03). The median length of hospital stay (LOS) was shorter in group A than in group P, 4 (2–8) days vs. 6 (3–10) days, but this was not statistically significant (p = 0.06)

#### Surgical outcomes

There was a difference in knee flexion between group A and group P at 24 h postoperatively; 90 (60–105) vs. 60 (30–85) degrees (p < 0.001) and 48 h: 75 (50–105) vs. 60 (40–90) degrees (p = 0.003) (Table 3), but no statistically significant differences were found at 3 days and 3 months postoperatively. No significant differences were found between the groups in knee extension, except at discharge. The TUG-test did not show any differences between the groups postoperatively.

**Table 3.** Mobilization and patient satisfaction

Outcome	Group A median (range)	n	Group P median (range)	n	p-value
Knee extension (degrees)					
Preop.	5 (0–30)	23	5 (0–20)	19	
24 h postop.	10 (0–20)	24	10 (0–20)	19	0.4
48 h postop.	10 (0–15)	23	10 (0–15)	21	0.4
Discharge	10 (0–10)	24	10 (5–10)	23	0.01
3 days postop.	10 (0–15)	22	10 (0–15)	22	0.4
3 months postop.	5 (0–10)	20	5 (0–15)	23	0.6
Knee flexion (degrees)					
Preop.	120 (90–153)	23	120 (100–135)	19	
24 h postop.	90 (60–105)	24	60 (30–85)	23	< 0.001
48 h postop.	75 (50–105)	23	60 (40–90)	22	0.003
Discharge	85 (60–115)	24	75 (60–100)	23	0.2
3 days postop.	75 (60–110)	22	62 (45–90)	22	0.09
3 months postop.	112 (100–125)	20	110 (90–130)	22	0.3
TUG test (seconds)					
Preop.	10 (6–26)	21	9 (6–28)	17	
48 h postop.	22 (10–51)	15	27 (8–35)	8	na **^a^**
3 days postop.	18 (9–49)	16	20 (8–80)	12	na **^a^**
7 days postop.	18 (10–48)	24	17 (7–65)	21	0.8
14 days postop.	14 (7–42)	22	10 (6–25)	22	0.5
3 months postop	8 (6–16)	20	7 (5–19)	23	0.8
Patient satisfaction					
1 day postop.	4 (2–4)	23	3 (1–4)	20	< 0.001
7 days postop.	3 (1–4)	23	3 (1–4)	20	0.02 **^b^**
Oxford knee score					
Preop.	39 (26–49)	23	40 (27–42)	20	
14 days postop.	33 (18–42)	20	32 (25–45)	21	0.9
3 months postop.	16 (12–37)	20	16 (12–28)	23	0.8
EQ-5D					
Preop	0.66 (0.06–0.80)	23	0.20 (0.03–0.73)	23	
3 months postop.	1 (0.59–1)	21	1 (0.69–1)	23	1

n: number of patients who participated varied depending on patients' ability to cooperate.

TUG test: Timed up and go test.

^**a**^ na: not applicable. No statistical calculations were done due to the small number of patients in each group.

^**b**^ Patient satisfaction: Excellent = 4, good = 3, inadequate = 2, poor = 1. In group A, 20/23 scored 3–4 as compared to 12/20 in group P 7 days postoperatively.

Oxford knee score: 12 (the best possible) to 60 (the worst possible).

EQ-5D health outcome: 1 = perfect health; 0 = poor health.

Patient satisfaction scores differed between the groups on day 1 (p < 0.001) and on day 7 (p = 0.02). Oxford Knee Score and EQ-5D value were similar postoperatively, at 14 days, and at 3 month in both groups (Table 3).

#### Adverse effects

There were no major surgical complications. There was a lower incidence of nausea, pruritus, and sedation in group A than in group P (Table 4). There were 3 positive cultures from the catheter tips, all with isolated coagulase-negative Staphylococcus, 2 in group A and 1 in group P. No antibiotics were given and no clinical signs of infection were found during the follow-up period. None of the patients were re-admitted to the hospital for any adverse effects or complications during the 3-month follow-up period. We did not find any deep venous thrombosis or insufficient wound heeling.

**Table 4.** Side effects. Values are number of patients in each category

	Group A (n = 24)	Group P (n = 23)	p-value
Nausea			
0–24 h	9	16	0.03
24–48 h	4	8	0.2
Vomiting			
0–24 h	5	9	0.2
24–48 h	1	3	0.4
Pruritus			
0–24 h	1	9	0.004
24–48 h	0	6	0.009
Sedation			
0–24 h	0	5	0.02
24–48 h	0	1	0.5

#### Plasma concentrations of ropivacaine

The individual maximum unbound venous plasma concentration of ropivacaine varied from 0.032 μg/mL to 0.12 μg/mL in the subgroup of 8 patients studied. None of these patients showed any clinical evidence of systemic LA toxicity.

## Discussion

The total consumption of analgesics was lower in the LIA group than in the placebo group, which was our primary endpoint, during the 48-h test period. This is consistent with our previous study on unicompartmental knee surgery (Essving et al. 2009) and also the findings of others using a similar (but modified) technique during TKA (Busch et al. 2006, Vendittoli et al. 2006, Andersen et al. 2008). Although TKA would be expected to be more painful than unicompartmental knee surgery, we found that both analgesic consumption and pain intensity in the LIA groups were similar between the studies. This may be because we increased the dose of ropivacaine injected periarticularly from 200 mg to 400 mg in the present study. The pain intensity measured by VAS was generally lower in the LIA group than in the placebo group during the first 48 h, specifically pain on movement. This would further confirm that local infiltration of analgesics periarticularly is efficacious and can therefore be recommended for analgesia following TKA. However, does better postoperative analgesia also translate into improved patient outcome?

We measured several patient outcomes over a 3-month period in order to determine whether improved analgesia also gives better outcome for the patient. Thus, patient satisfaction, which is a direct—though crude—measure of a subjective feeling of quality of care, was higher in patients in the LIA group on days 1 and 7 postoperatively. Surgical outcome, measured as the degree of knee flexion postoperatively, was also greater in the LIA group than in the placebo group, which could have been an indirect result of better analgesia in this group of patients. Outcome measures were better in the LIA group only in the early period, however, before home discharge. Taken together, these results indicate that patients receiving local infiltration analgesia feel subjectively better and are objectively better in the early postoperative period than those not receiving it. Thus, the next question is whether these improved patient outcomes also lead to cost reduction for the healthcare system.

Although we did not measure direct costs for patients or for the healthcare system, we did measure the time taken to achieve discharge criteria and also length of hospital stay. We found that time to home readiness in the LIA group was shorter than in the placebo group by 2 days, a statistically significant and a clinically relevant reduction. The actual length of hospital stay was shorter, but the difference did not reach statistical significance and was similar to the results of other authors using a similar technique in patients undergoing TKA (Busch et al. 2006, Vendittoli et al. 2006, Toftdahl et al. 2007). Although home readiness can be affected by several factors, we used objective and clearly defined discharge criteria that we have described previously (Essving et al. 2009). In addition, the personnel determining home readiness were blinded regarding the treatment arm, thus reducing bias. The slightly longer length of hospital stay compared to the time to achieve discharge criteria was the result of non-medical considerations such as the absence of a relative to take care of the patient at home or delay in hospital discharge for administrative reasons.

Although there were several outcome variables that were in favor of the LIA technique, we did not find any statistically significant differences in certain other parameters. Thus, there were no differences between the groups in the TUG test, the Oxford knee score, or in the EQ5D at the measurement points described earlier. There could be several reasons for the absence of such differences. Health-related quality of life as determined by the EQ5D and Oxford knee score is a crude measure of ADL function, and is therefore heavily weighed down by factors other than early postoperative pain. Thus, when we measured these parameters after 14 days, there was no difference in pain intensity between the groups; thus, we did not find any differences in the EQ5D and the Oxford knee score.

An important question is whether a catheter should be placed intraarticularly, exposing the patient to the risk of infection. In order to answer this question, a risk-benefit analysis must be done. The benefit that the patient obtained was the possibility of re-injection of analgesic drugs after 21 h in order to prolong analgesia and improve patient outcome. The pain relief achieved as a consequence of re-injection the following day after the operation was statistically significant and clinically relevant when compared to pain intensity between the groups, specifically pain on movement—a benefit that persisted for up to 48 h. This, taken together with the fact that morphine consumption was considerably reduced in the LIA group from 24 h to 48 h would suggest that the catheter prolonged the analgesia after the first day. However, it would be interesting to study this benefit longitudinally to better understand the benefits of leaving the catheter in situ after the operation. There was no evidence of any postoperative infections or infection after a 3-month follow-up, which indicates that the risk of infection, if any, was small. However, this study is small and more studies should be done in order to evaluate the risk of infection. It is also important to emphasize that in our study, the catheters were placed under aseptic conditions by the surgeon at the end of the operation; bacterial filters were used, which is a routine with other catheter techniques, and the investigators who injected drugs after 21 h did so under aseptic conditions. We believe that, if appropriately and correctly done, the benefit of retaining a catheter intraarticularly outweighs the potential risk of complications and until proved otherwise, catheters may be inserted into the knee joint following TKA, albeit under antibiotic protection.

Another question that arises is whether the dose of LA injected periarticularly may produce toxic concentrations through systemic absorption. Indeed, we did inject higher doses than in our previous study due to the more extensive surgery. The total dose of ropivacaine injected was, however, below the maximum dose recommended by the manufacturers. Furthermore, we confirmed that the maximum concentration of LA seen in any of the 8 patients in whom plasma concentration was measured was far below the known toxic concentrations in humans (Knudsen et al. 1997) and corresponded well with findings from other authors (Busch et al. 2006). In addition, none of the patients had any evidence of systemic LA toxicity, confirming that these doses are safe when injected periarticularly.

Finally, the safety of ketorolac injected intraarticularly can be questioned in view of some evidence, specifically from animal studies, of the risk of delay in bone healing (Meunier and Aspenberg 2006). We cannot answer this question, as the follow-up period was only 3 months in our patients.

In summary, our findings provide strong evidence that the LIA technique is effective and safe, specifically for pain management in the early postoperative period, and it can therefore be recommended following TKA. Future studies should focus on the drugs and doses that should be used in the LIA mixture and on the length of time that the catheter should remain in situ.
